# 4-[4-(4-Chloro­benzo­yl)-2,3-diphenyl­isoxazolidin-5-yl]-1-(4-meth­oxy­phen­yl)-3-phenyl­azetidin-2-one

**DOI:** 10.1107/S1600536812038123

**Published:** 2012-09-12

**Authors:** Sivasubramanian Suhitha, Thothadri Srinivasan, Ramanathan Prasanna, Raghavachary Raghunathan, Devadasan Velmurugan

**Affiliations:** a Centre of Advanced Study in Crystallography and Biophysics, University of Madras, Guindy Campus, Chennai 600 025, India; b Department of Organic Chemistry, University of Madras, Guindy Campus, Chennai 600 025, India

## Abstract

In the title compound, C_38_H_31_ClN_2_O_4_, the isoxazole ring adopts an envelope conformation with the N atom as the flap. The crystal packing is stabilized by C—H⋯O hydrogen bonds, forming chains running along the *c*-axis direction.

## Related literature

For general background to β-lactams, see: Jones *et al.* (1989 ▶); Brakhage (1998 ▶); Banik & Becker (2000 ▶). For a related structure, see: Sundaramoorthy *et al.* (2012 ▶).
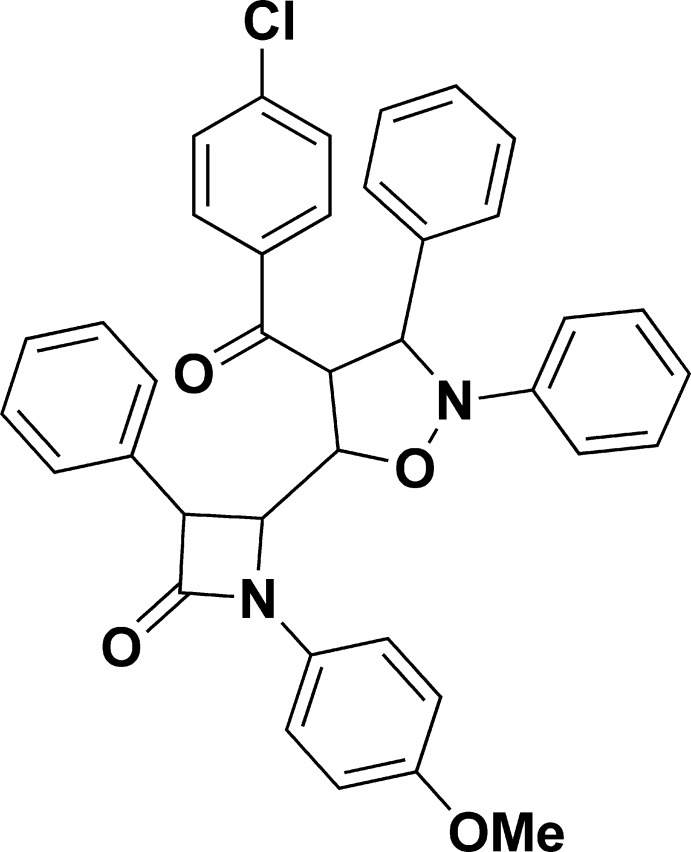


## Experimental

### 

#### Crystal data

C_38_H_31_ClN_2_O_4_
*M* *_r_* = 615.10
Monoclinic, 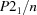

*a* = 10.1221 (10) Å
*b* = 17.4890 (17) Å
*c* = 17.7421 (18) Å
β = 98.150 (6)°
*V* = 3109.1 (5) Å^3^
*Z* = 4
Mo *K*α radiation
μ = 0.17 mm^−1^
*T* = 293 K
0.30 × 0.25 × 0.20 mm

#### Data collection

Bruker SMART APEXII area-detector diffractometer
Absorption correction: multi-scan (*SADABS*; Bruker, 2008 ▶) *T*_min_ = 0.951, *T*_max_ = 0.967
23486 measured reflections
5491 independent reflections
3214 reflections with *I* > 2σ(*I*)
*R*_int_ = 0.050

#### Refinement

*R*[*F* ^2^ > 2σ(*F* ^2^)] = 0.044
*wR*(*F* ^2^) = 0.132
*S* = 0.99
5491 reflections
407 parameters
H-atom parameters constrained
Δρ_max_ = 0.29 e Å^−3^
Δρ_min_ = −0.41 e Å^−3^

### 

Data collection: *APEX2* (Bruker, 2008 ▶); cell refinement: *SAINT* (Bruker, 2008 ▶); data reduction: *SAINT*; program(s) used to solve structure: *SHELXS97* (Sheldrick, 2008 ▶); program(s) used to refine structure: *SHELXL97* (Sheldrick, 2008 ▶); molecular graphics: *ORTEP-3* (Farrugia, 1997 ▶); software used to prepare material for publication: *SHELXL97* and *PLATON* (Spek, 2009 ▶).

## Supplementary Material

Crystal structure: contains datablock(s) global, I. DOI: 10.1107/S1600536812038123/bt6829sup1.cif

Structure factors: contains datablock(s) I. DOI: 10.1107/S1600536812038123/bt6829Isup2.hkl

Supplementary material file. DOI: 10.1107/S1600536812038123/bt6829Isup3.cml

Additional supplementary materials:  crystallographic information; 3D view; checkCIF report

## Figures and Tables

**Table 1:** Hydrogen-bond geometry (Å, °)

*D*—H⋯*A*	*D*—H	H⋯*A*	*D*⋯*A*	*D*—H⋯*A*
C19—H19⋯O2^i^	0.98	2.41	3.338 (3)	158
C21—H21⋯O2^i^	0.93	2.50	3.425 (4)	173
C37—H37⋯O1^ii^	0.93	2.58	3.407 (4)	148

Symmetry codes: (i) 
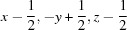
; (ii) 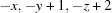.

**Fig. 1.**
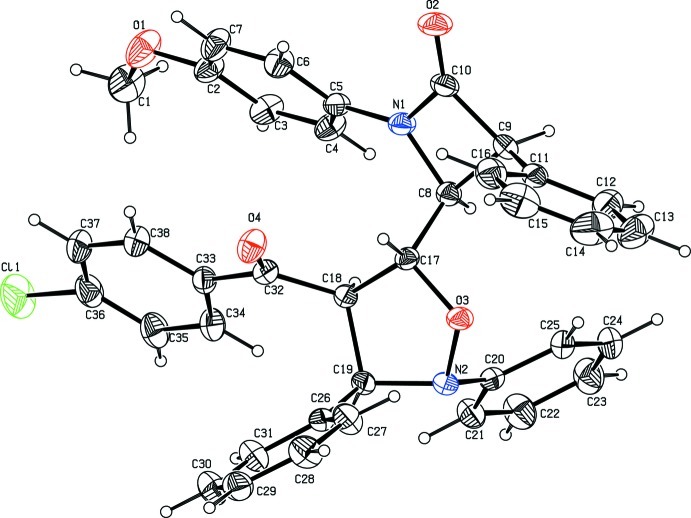
The molecular structure of the title compound, showing displacement ellipsoids drawn at the 30% probability level. H atoms are presented as small spheres of arbitrary radius.

**Fig. 2.**
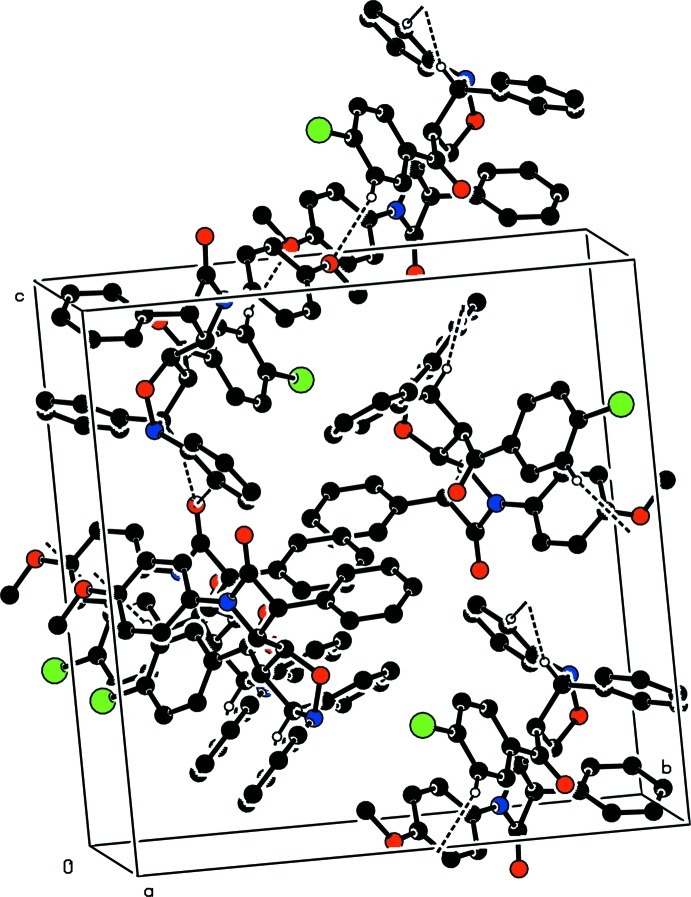
The crystal packing of the title compound viewed down the c axis. H-atoms not involved in H-bonds have been excluded for clarity.

## supplementary crystallographic information

### Comment

The role of β-lactam antibiotics is well known (Banik & Becker, 2000). The most commonly used β-lactam antibiotics for the therapy of infectious diseases are penicillin and cephalosporin (Brakhage, 1998). β-Lactam based antibiotics have been successfully used in the treatment of infectious diseases for many years (Jones *et al.*, 1989). In view of potential applications, the crystal structure determination of the titled β-lactam derivative was carried out.

In the title compound (Fig. 1), the β-lactam ring makes a dihedral angle of 10.33 (14)° with a isoxazole ring (N2/03/C17/C18/C19), a dihedral angle of 40.33 (14)° with the methoxy phenyl ring and a dihedral angle of 70.05 (16)° with the unsubstited phenyl ring. The dihedral angle between the isoxazole ring and the unsubstited phenyl ring (C20/C21/C22/C23/C24/C25) is 81.42 (12)°, which shows that they are almost orthogonal to each other. The dihedral angle between the isoxazole ring and the unsubstited phenyl ring (C11/C12/C13/C14/C15/C16) is 80.38 (14)°, which shows that they are also almost orthogonal to each other. The isoxazole ring makes a dihedral angle of 40.35 (12)° with the chlorolophenyl ring.

The oxygen atom attached to the β-lactam ring deviates by -0.1871 (18)Å. The oxygen atom attached to the phenyl ring deviates by 0.0237 (21)Å. The packing of the crystal structure is stabilized by intermolecular C—H···O hydrogen bonds (Fig. 2). A related structure was published by Sundaramoorthy *et al.* (2012).

### Experimental

To a solution of the N-benzylideneaniline oxide (1 mol) in dry acetonitrile (20 ml) was added (E)-4-(3-(4-chlorophenyl)-3-oxoprop-1-enyl) -1-(4-methoxyphenyl)-3-phenylazetidin-2-one (1 mol) under N_2_ atmosphere. The reaction was refluxed for 48 hours. After the completion of the reaction the solvent was distilled off under reduced pressure and the crude product was purified by column chromatography using 9:1 mixture of hexane-ethyl acetate. Crystallization of the pure compound was done using 1:1 mixture of chloroform-ethyl acetate.

### Refinement

The hydrogen atoms were placed in calculated positions with C—H = 0.93 Å to 0.98 Å and refined using a riding model with fixed isotropic displacement parameters: *U*_iso_(H) = 1.5*U*_eq_(C) for the methyl group and *U*_iso_(H) = 1.2*U*_eq_(C) for the remaining H atoms.

### Crystal data

**Table:**

C_38_H_31_ClN_2_O_4_	*F*(000) = 1288
*M**_r_* = 615.10	*D*_x_ = 1.314 Mg m^−^^3^
Monoclinic, *P*2_1_/*n*	Mo *K*α radiation, λ = 0.71073 Å
Hall symbol: -P 2yn	Cell parameters from 7716 reflections
*a* = 10.1221 (10) Å	θ = 1.6–28.4°
*b* = 17.4890 (17) Å	µ = 0.17 mm^−^^1^
*c* = 17.7421 (18) Å	*T* = 293 K
β = 98.150 (6)°	Block, colourless
*V* = 3109.1 (5) Å^3^	0.30 × 0.25 × 0.20 mm
*Z* = 4	

### Data collection

**Table:**

Bruker SMART APEXII area-detector diffractometer	5491 independent reflections
Radiation source: fine-focus sealed tube	3214 reflections with *I* > 2σ(*I*)
Graphite monochromator	*R*_int_ = 0.050
ω and φ scans	θ_max_ = 25.1°, θ_min_ = 1.6°
Absorption correction: multi-scan (*SADABS*; Bruker, 2008)	*h* = −12→11
*T*_min_ = 0.951, *T*_max_ = 0.967	*k* = −20→19
23486 measured reflections	*l* = −21→17

### Refinement

**Table:**

Refinement on *F*^2^	Primary atom site location: structure-invariant direct methods
Least-squares matrix: full	Secondary atom site location: difference Fourier map
*R*[*F*^2^ > 2σ(*F*^2^)] = 0.044	Hydrogen site location: inferred from neighbouring sites
*wR*(*F*^2^) = 0.132	H-atom parameters constrained
*S* = 0.99	*w* = 1/[σ^2^(*F*_o_^2^) + (0.0566*P*)^2^ + 0.7516*P*] where *P* = (*F*_o_^2^ + 2*F*_c_^2^)/3
5491 reflections	(Δ/σ)_max_ < 0.001
407 parameters	Δρ_max_ = 0.29 e Å^−^^3^
0 restraints	Δρ_min_ = −0.41 e Å^−^^3^

### Special details

**Table:**

Geometry. All esds (except the esd in the dihedral angle between two l.s. planes) are estimated using the full covariance matrix. The cell esds are taken into account individually in the estimation of esds in distances, angles and torsion angles; correlations between esds in cell parameters are only used when they are defined by crystal symmetry. An approximate (isotropic) treatment of cell esds is used for estimating esds involving l.s. planes.
Refinement. Refinement of F^2^ against ALL reflections. The weighted R-factor wR and goodness of fit S are based on F^2^, conventional R-factors R are based on F, with F set to zero for negative F^2^. The threshold expression of F^2^ > 2sigma(F^2^) is used only for calculating R-factors(gt) etc. and is not relevant to the choice of reflections for refinement. R-factors based on F^2^ are statistically about twice as large as those based on F, and R- factors based on ALL data will be even larger.

### Fractional atomic coordinates and isotropic or equivalent isotropic displacement parameters (Å^2^)

**Table:**

	*x*	*y*	*z*	*U*_iso_*/*U*_eq_	
C1	0.1235 (4)	0.56910 (18)	0.92991 (18)	0.0952 (11)	
H1A	0.1944	0.6030	0.9211	0.143*	
H1B	0.0490	0.5985	0.9417	0.143*	
H1C	0.0966	0.5391	0.8851	0.143*	
C2	0.2637 (3)	0.46709 (15)	0.98273 (15)	0.0641 (7)	
C3	0.3357 (3)	0.46562 (15)	0.92296 (15)	0.0671 (8)	
H3	0.3218	0.5028	0.8852	0.080*	
C4	0.4285 (3)	0.40889 (15)	0.91917 (14)	0.0623 (7)	
H4	0.4782	0.4085	0.8789	0.075*	
C5	0.4491 (2)	0.35290 (13)	0.97360 (13)	0.0490 (6)	
C6	0.3775 (3)	0.35466 (16)	1.03403 (14)	0.0639 (7)	
H6	0.3914	0.3174	1.0718	0.077*	
C7	0.2856 (3)	0.41155 (17)	1.03833 (15)	0.0748 (8)	
H7	0.2374	0.4127	1.0792	0.090*	
C8	0.5786 (2)	0.24964 (13)	0.90286 (12)	0.0473 (6)	
H8	0.6073	0.2821	0.8632	0.057*	
C9	0.6982 (2)	0.21662 (14)	0.95941 (13)	0.0531 (6)	
H9	0.7803	0.2432	0.9515	0.064*	
C10	0.6345 (2)	0.25842 (15)	1.02071 (14)	0.0567 (6)	
C11	0.7257 (2)	0.13271 (15)	0.96688 (13)	0.0549 (6)	
C12	0.8284 (3)	0.10087 (19)	0.93330 (15)	0.0781 (9)	
H12	0.8795	0.1319	0.9062	0.094*	
C13	0.8558 (4)	0.0236 (2)	0.9396 (2)	0.1023 (12)	
H13	0.9246	0.0029	0.9166	0.123*	
C14	0.7824 (5)	−0.0220 (2)	0.9795 (2)	0.1094 (13)	
H14	0.8021	−0.0738	0.9842	0.131*	
C15	0.6803 (4)	0.0074 (2)	1.0126 (2)	0.0951 (10)	
H15	0.6295	−0.0244	1.0392	0.114*	
C16	0.6521 (3)	0.08535 (18)	1.00663 (16)	0.0729 (8)	
H16	0.5829	0.1055	1.0298	0.087*	
C17	0.4693 (2)	0.19375 (13)	0.87231 (12)	0.0433 (5)	
H17	0.4398	0.1668	0.9154	0.052*	
C18	0.3478 (2)	0.23073 (13)	0.82526 (11)	0.0426 (5)	
H18	0.3732	0.2817	0.8093	0.051*	
C19	0.3247 (2)	0.17755 (14)	0.75339 (12)	0.0470 (6)	
H19	0.2896	0.2083	0.7089	0.056*	
C20	0.5419 (2)	0.20436 (14)	0.71008 (13)	0.0490 (6)	
C21	0.4897 (3)	0.25163 (16)	0.65020 (14)	0.0638 (7)	
H21	0.3981	0.2527	0.6341	0.077*	
C22	0.5739 (3)	0.29695 (18)	0.61468 (17)	0.0814 (9)	
H22	0.5386	0.3287	0.5748	0.098*	
C23	0.7086 (3)	0.29552 (19)	0.63755 (19)	0.0838 (9)	
H23	0.7648	0.3266	0.6138	0.101*	
C24	0.7602 (3)	0.24798 (18)	0.69571 (17)	0.0747 (8)	
H24	0.8520	0.2467	0.7110	0.090*	
C25	0.6786 (2)	0.20225 (16)	0.73171 (14)	0.0608 (7)	
H25	0.7151	0.1698	0.7707	0.073*	
C26	0.2310 (2)	0.11091 (14)	0.75998 (12)	0.0483 (6)	
C27	0.2709 (3)	0.04226 (16)	0.79247 (16)	0.0697 (8)	
H27	0.3607	0.0343	0.8106	0.084*	
C28	0.1794 (3)	−0.01581 (17)	0.79888 (17)	0.0771 (8)	
H28	0.2083	−0.0619	0.8215	0.092*	
C29	0.0477 (3)	−0.00560 (18)	0.77219 (16)	0.0706 (8)	
H29	−0.0138	−0.0442	0.7769	0.085*	
C30	0.0075 (3)	0.06236 (19)	0.73840 (17)	0.0786 (8)	
H30	−0.0821	0.0699	0.7196	0.094*	
C31	0.0981 (3)	0.11970 (17)	0.73199 (15)	0.0685 (7)	
H31	0.0690	0.1653	0.7083	0.082*	
C32	0.2290 (2)	0.23887 (14)	0.86813 (13)	0.0489 (6)	
C33	0.1259 (2)	0.29713 (13)	0.84202 (14)	0.0522 (6)	
C34	0.1156 (2)	0.33164 (15)	0.77096 (16)	0.0642 (7)	
H34	0.1747	0.3178	0.7377	0.077*	
C35	0.0194 (3)	0.38610 (16)	0.74874 (19)	0.0758 (8)	
H35	0.0127	0.4082	0.7007	0.091*	
C36	−0.0656 (3)	0.40701 (16)	0.7980 (2)	0.0777 (9)	
C37	−0.0583 (3)	0.37409 (19)	0.8685 (2)	0.0842 (9)	
H37	−0.1172	0.3890	0.9015	0.101*	
C38	0.0370 (3)	0.31840 (16)	0.89081 (16)	0.0698 (8)	
H38	0.0412	0.2955	0.9384	0.084*	
N1	0.54326 (19)	0.29368 (11)	0.96857 (10)	0.0519 (5)	
N2	0.45940 (18)	0.15261 (11)	0.74562 (10)	0.0497 (5)	
O1	0.1687 (2)	0.52014 (13)	0.99171 (11)	0.0979 (7)	
O2	0.65372 (19)	0.26133 (11)	1.08976 (10)	0.0793 (6)	
O3	0.51966 (15)	0.13785 (9)	0.82304 (8)	0.0513 (4)	
O4	0.21907 (18)	0.19892 (11)	0.92295 (10)	0.0692 (5)	
Cl1	−0.18375 (10)	0.47664 (6)	0.77110 (7)	0.1308 (4)	

### Atomic displacement parameters (Å^2^)

**Table:**

	*U*^11^	*U*^22^	*U*^33^	*U*^12^	*U*^13^	*U*^23^
C1	0.113 (3)	0.078 (2)	0.091 (2)	0.039 (2)	0.005 (2)	0.0199 (19)
C2	0.0778 (18)	0.0607 (18)	0.0536 (16)	0.0226 (14)	0.0088 (14)	−0.0012 (14)
C3	0.096 (2)	0.0519 (17)	0.0545 (16)	0.0145 (15)	0.0133 (15)	0.0090 (13)
C4	0.0837 (19)	0.0566 (17)	0.0489 (15)	0.0082 (15)	0.0170 (13)	0.0030 (13)
C5	0.0548 (14)	0.0471 (15)	0.0430 (13)	0.0035 (12)	0.0000 (11)	−0.0054 (12)
C6	0.0723 (18)	0.0700 (18)	0.0498 (15)	0.0125 (15)	0.0099 (13)	0.0132 (14)
C7	0.084 (2)	0.091 (2)	0.0535 (16)	0.0277 (17)	0.0215 (14)	0.0107 (16)
C8	0.0481 (13)	0.0491 (14)	0.0438 (13)	0.0020 (11)	0.0033 (10)	−0.0010 (11)
C9	0.0403 (13)	0.0650 (17)	0.0508 (14)	−0.0004 (12)	−0.0042 (10)	−0.0040 (13)
C10	0.0537 (15)	0.0605 (17)	0.0512 (16)	0.0007 (12)	−0.0091 (12)	−0.0105 (13)
C11	0.0495 (14)	0.0650 (18)	0.0457 (14)	0.0104 (13)	−0.0084 (11)	−0.0058 (13)
C12	0.080 (2)	0.095 (2)	0.0580 (17)	0.0271 (18)	0.0027 (14)	−0.0005 (16)
C13	0.122 (3)	0.106 (3)	0.077 (2)	0.060 (3)	0.004 (2)	−0.007 (2)
C14	0.148 (4)	0.080 (3)	0.092 (3)	0.039 (3)	−0.014 (3)	−0.007 (2)
C15	0.112 (3)	0.075 (3)	0.091 (2)	0.000 (2)	−0.010 (2)	0.019 (2)
C16	0.0710 (19)	0.075 (2)	0.0696 (19)	0.0076 (16)	0.0004 (15)	0.0054 (16)
C17	0.0471 (13)	0.0475 (14)	0.0350 (11)	0.0047 (11)	0.0050 (9)	−0.0009 (11)
C18	0.0419 (12)	0.0458 (14)	0.0396 (12)	0.0019 (10)	0.0037 (9)	0.0001 (10)
C19	0.0415 (12)	0.0592 (15)	0.0387 (12)	0.0030 (11)	0.0005 (9)	0.0007 (11)
C20	0.0489 (14)	0.0538 (15)	0.0451 (13)	−0.0039 (12)	0.0098 (11)	−0.0106 (12)
C21	0.0522 (15)	0.0765 (19)	0.0610 (16)	−0.0079 (14)	0.0027 (12)	0.0081 (15)
C22	0.073 (2)	0.093 (2)	0.079 (2)	−0.0151 (17)	0.0104 (16)	0.0232 (18)
C23	0.069 (2)	0.090 (2)	0.096 (2)	−0.0198 (17)	0.0265 (18)	0.008 (2)
C24	0.0476 (15)	0.094 (2)	0.084 (2)	−0.0071 (15)	0.0167 (15)	−0.0120 (19)
C25	0.0465 (14)	0.0752 (19)	0.0613 (16)	0.0064 (13)	0.0101 (12)	−0.0069 (14)
C26	0.0502 (14)	0.0548 (16)	0.0397 (13)	−0.0016 (12)	0.0052 (10)	−0.0042 (12)
C27	0.0583 (16)	0.0639 (19)	0.084 (2)	−0.0020 (15)	−0.0022 (14)	−0.0003 (16)
C28	0.083 (2)	0.0572 (19)	0.089 (2)	−0.0065 (16)	0.0047 (17)	0.0030 (16)
C29	0.0679 (19)	0.076 (2)	0.0694 (18)	−0.0208 (16)	0.0155 (15)	−0.0064 (16)
C30	0.0526 (16)	0.091 (2)	0.090 (2)	−0.0152 (17)	0.0039 (15)	0.0075 (19)
C31	0.0534 (16)	0.077 (2)	0.0726 (18)	−0.0081 (14)	−0.0015 (13)	0.0103 (15)
C32	0.0526 (14)	0.0484 (15)	0.0466 (14)	−0.0005 (11)	0.0094 (11)	−0.0017 (12)
C33	0.0432 (13)	0.0490 (15)	0.0646 (16)	0.0015 (11)	0.0087 (11)	−0.0039 (13)
C34	0.0509 (15)	0.0632 (18)	0.0787 (19)	0.0096 (13)	0.0103 (13)	0.0086 (15)
C35	0.0585 (17)	0.0674 (19)	0.099 (2)	0.0093 (15)	0.0019 (16)	0.0116 (17)
C36	0.0550 (17)	0.0597 (19)	0.114 (3)	0.0115 (14)	−0.0048 (17)	−0.0134 (19)
C37	0.0585 (18)	0.089 (2)	0.107 (3)	0.0188 (17)	0.0163 (17)	−0.027 (2)
C38	0.0583 (16)	0.077 (2)	0.0760 (18)	0.0093 (15)	0.0149 (14)	−0.0115 (16)
N1	0.0569 (12)	0.0550 (13)	0.0407 (11)	0.0073 (10)	−0.0040 (9)	−0.0063 (10)
N2	0.0462 (11)	0.0620 (13)	0.0387 (10)	0.0027 (10)	−0.0020 (8)	−0.0027 (9)
O1	0.1229 (18)	0.1013 (17)	0.0726 (13)	0.0604 (15)	0.0244 (12)	0.0142 (12)
O2	0.0865 (13)	0.0973 (15)	0.0458 (11)	0.0217 (11)	−0.0194 (9)	−0.0177 (10)
O3	0.0540 (9)	0.0556 (10)	0.0408 (8)	0.0124 (8)	−0.0055 (7)	−0.0049 (8)
O4	0.0768 (12)	0.0724 (13)	0.0636 (11)	0.0104 (10)	0.0279 (9)	0.0149 (10)
Cl1	0.0894 (7)	0.1048 (8)	0.1876 (11)	0.0526 (6)	−0.0169 (6)	−0.0139 (7)

### Geometric parameters (Å, º)

**Table:**

C1—O1	1.415 (3)	C18—H18	0.9800
C1—H1A	0.9600	C19—N2	1.457 (3)
C1—H1B	0.9600	C19—C26	1.517 (3)
C1—H1C	0.9600	C19—H19	0.9800
C2—O1	1.362 (3)	C20—C25	1.383 (3)
C2—C3	1.369 (3)	C20—C21	1.390 (3)
C2—C7	1.380 (4)	C20—N2	1.436 (3)
C3—C4	1.375 (3)	C21—C22	1.380 (4)
C3—H3	0.9300	C21—H21	0.9300
C4—C5	1.370 (3)	C22—C23	1.367 (4)
C4—H4	0.9300	C22—H22	0.9300
C5—C6	1.377 (3)	C23—C24	1.370 (4)
C5—N1	1.419 (3)	C23—H23	0.9300
C6—C7	1.372 (4)	C24—C25	1.371 (4)
C6—H6	0.9300	C24—H24	0.9300
C7—H7	0.9300	C25—H25	0.9300
C8—N1	1.483 (3)	C26—C27	1.368 (3)
C8—C17	1.518 (3)	C26—C31	1.375 (3)
C8—C9	1.569 (3)	C27—C28	1.390 (4)
C8—H8	0.9800	C27—H27	0.9300
C9—C11	1.496 (3)	C28—C29	1.362 (4)
C9—C10	1.527 (3)	C28—H28	0.9300
C9—H9	0.9800	C29—C30	1.367 (4)
C10—O2	1.214 (3)	C29—H29	0.9300
C10—N1	1.359 (3)	C30—C31	1.375 (4)
C11—C16	1.374 (4)	C30—H30	0.9300
C11—C12	1.386 (4)	C31—H31	0.9300
C12—C13	1.381 (4)	C32—O4	1.213 (3)
C12—H12	0.9300	C32—C33	1.485 (3)
C13—C14	1.355 (5)	C33—C38	1.385 (3)
C13—H13	0.9300	C33—C34	1.388 (3)
C14—C15	1.360 (5)	C34—C35	1.379 (4)
C14—H14	0.9300	C34—H34	0.9300
C15—C16	1.394 (4)	C35—C36	1.360 (4)
C15—H15	0.9300	C35—H35	0.9300
C16—H16	0.9300	C36—C37	1.370 (4)
C17—O3	1.451 (2)	C36—Cl1	1.726 (3)
C17—C18	1.529 (3)	C37—C38	1.389 (4)
C17—H17	0.9800	C37—H37	0.9300
C18—C32	1.518 (3)	C38—H38	0.9300
C18—C19	1.569 (3)	N2—O3	1.445 (2)
			
O1—C1—H1A	109.5	N2—C19—C18	102.75 (16)
O1—C1—H1B	109.5	C26—C19—C18	114.55 (17)
H1A—C1—H1B	109.5	N2—C19—H19	109.0
O1—C1—H1C	109.5	C26—C19—H19	109.0
H1A—C1—H1C	109.5	C18—C19—H19	109.0
H1B—C1—H1C	109.5	C25—C20—C21	119.0 (2)
O1—C2—C3	124.4 (2)	C25—C20—N2	118.8 (2)
O1—C2—C7	116.1 (2)	C21—C20—N2	121.9 (2)
C3—C2—C7	119.4 (2)	C22—C21—C20	119.9 (2)
C2—C3—C4	119.6 (2)	C22—C21—H21	120.1
C2—C3—H3	120.2	C20—C21—H21	120.1
C4—C3—H3	120.2	C23—C22—C21	120.6 (3)
C5—C4—C3	121.2 (2)	C23—C22—H22	119.7
C5—C4—H4	119.4	C21—C22—H22	119.7
C3—C4—H4	119.4	C22—C23—C24	119.5 (3)
C4—C5—C6	119.3 (2)	C22—C23—H23	120.2
C4—C5—N1	120.9 (2)	C24—C23—H23	120.2
C6—C5—N1	119.8 (2)	C23—C24—C25	120.9 (3)
C7—C6—C5	119.7 (2)	C23—C24—H24	119.5
C7—C6—H6	120.2	C25—C24—H24	119.5
C5—C6—H6	120.2	C24—C25—C20	120.0 (3)
C6—C7—C2	120.8 (2)	C24—C25—H25	120.0
C6—C7—H7	119.6	C20—C25—H25	120.0
C2—C7—H7	119.6	C27—C26—C31	117.7 (2)
N1—C8—C17	111.74 (18)	C27—C26—C19	123.8 (2)
N1—C8—C9	86.49 (16)	C31—C26—C19	118.4 (2)
C17—C8—C9	116.66 (19)	C26—C27—C28	121.0 (3)
N1—C8—H8	113.1	C26—C27—H27	119.5
C17—C8—H8	113.1	C28—C27—H27	119.5
C9—C8—H8	113.1	C29—C28—C27	120.4 (3)
C11—C9—C10	119.9 (2)	C29—C28—H28	119.8
C11—C9—C8	122.33 (19)	C27—C28—H28	119.8
C10—C9—C8	85.07 (17)	C28—C29—C30	118.8 (3)
C11—C9—H9	109.1	C28—C29—H29	120.6
C10—C9—H9	109.1	C30—C29—H29	120.6
C8—C9—H9	109.1	C29—C30—C31	120.7 (3)
O2—C10—N1	131.6 (2)	C29—C30—H30	119.7
O2—C10—C9	135.7 (2)	C31—C30—H30	119.7
N1—C10—C9	92.71 (19)	C30—C31—C26	121.3 (3)
C16—C11—C12	118.2 (3)	C30—C31—H31	119.4
C16—C11—C9	121.9 (2)	C26—C31—H31	119.4
C12—C11—C9	119.8 (3)	O4—C32—C33	120.7 (2)
C13—C12—C11	120.8 (3)	O4—C32—C18	120.6 (2)
C13—C12—H12	119.6	C33—C32—C18	118.7 (2)
C11—C12—H12	119.6	C38—C33—C34	118.6 (2)
C14—C13—C12	120.0 (3)	C38—C33—C32	118.6 (2)
C14—C13—H13	120.0	C34—C33—C32	122.7 (2)
C12—C13—H13	120.0	C35—C34—C33	121.3 (3)
C13—C14—C15	120.7 (4)	C35—C34—H34	119.4
C13—C14—H14	119.7	C33—C34—H34	119.4
C15—C14—H14	119.7	C36—C35—C34	119.2 (3)
C14—C15—C16	119.7 (4)	C36—C35—H35	120.4
C14—C15—H15	120.1	C34—C35—H35	120.4
C16—C15—H15	120.1	C35—C36—C37	121.1 (3)
C11—C16—C15	120.6 (3)	C35—C36—Cl1	119.3 (3)
C11—C16—H16	119.7	C37—C36—Cl1	119.6 (3)
C15—C16—H16	119.7	C36—C37—C38	120.0 (3)
O3—C17—C8	110.41 (17)	C36—C37—H37	120.0
O3—C17—C18	106.27 (16)	C38—C37—H37	120.0
C8—C17—C18	114.33 (18)	C33—C38—C37	119.8 (3)
O3—C17—H17	108.6	C33—C38—H38	120.1
C8—C17—H17	108.6	C37—C38—H38	120.1
C18—C17—H17	108.6	C10—N1—C5	133.33 (19)
C32—C18—C17	113.52 (18)	C10—N1—C8	94.76 (18)
C32—C18—C19	115.19 (17)	C5—N1—C8	131.89 (18)
C17—C18—C19	102.05 (17)	C20—N2—O3	109.67 (16)
C32—C18—H18	108.6	C20—N2—C19	117.59 (18)
C17—C18—H18	108.6	O3—N2—C19	103.78 (15)
C19—C18—H18	108.6	C2—O1—C1	118.5 (2)
N2—C19—C26	112.38 (19)	N2—O3—C17	108.38 (15)
			
O1—C2—C3—C4	−179.4 (3)	C18—C19—C26—C31	93.9 (3)
C7—C2—C3—C4	0.0 (4)	C31—C26—C27—C28	−1.8 (4)
C2—C3—C4—C5	1.0 (4)	C19—C26—C27—C28	178.0 (2)
C3—C4—C5—C6	−1.5 (4)	C26—C27—C28—C29	0.5 (4)
C3—C4—C5—N1	178.8 (2)	C27—C28—C29—C30	0.6 (4)
C4—C5—C6—C7	0.9 (4)	C28—C29—C30—C31	−0.5 (4)
N1—C5—C6—C7	−179.4 (2)	C29—C30—C31—C26	−0.8 (4)
C5—C6—C7—C2	0.1 (4)	C27—C26—C31—C30	1.9 (4)
O1—C2—C7—C6	178.9 (3)	C19—C26—C31—C30	−177.9 (2)
C3—C2—C7—C6	−0.6 (5)	C17—C18—C32—O4	−21.7 (3)
N1—C8—C9—C11	129.4 (2)	C19—C18—C32—O4	95.4 (3)
C17—C8—C9—C11	16.8 (3)	C17—C18—C32—C33	157.8 (2)
N1—C8—C9—C10	6.87 (17)	C19—C18—C32—C33	−85.1 (3)
C17—C8—C9—C10	−105.7 (2)	O4—C32—C33—C38	15.7 (4)
C11—C9—C10—O2	47.9 (4)	C18—C32—C33—C38	−163.8 (2)
C8—C9—C10—O2	172.6 (3)	O4—C32—C33—C34	−164.9 (2)
C11—C9—C10—N1	−132.2 (2)	C18—C32—C33—C34	15.6 (3)
C8—C9—C10—N1	−7.50 (18)	C38—C33—C34—C35	0.0 (4)
C10—C9—C11—C16	27.0 (3)	C32—C33—C34—C35	−179.4 (2)
C8—C9—C11—C16	−77.3 (3)	C33—C34—C35—C36	1.0 (4)
C10—C9—C11—C12	−152.7 (2)	C34—C35—C36—C37	−1.0 (5)
C8—C9—C11—C12	103.0 (3)	C34—C35—C36—Cl1	178.6 (2)
C16—C11—C12—C13	0.1 (4)	C35—C36—C37—C38	0.1 (5)
C9—C11—C12—C13	179.8 (3)	Cl1—C36—C37—C38	−179.6 (2)
C11—C12—C13—C14	−0.4 (5)	C34—C33—C38—C37	−1.0 (4)
C12—C13—C14—C15	0.9 (6)	C32—C33—C38—C37	178.4 (2)
C13—C14—C15—C16	−1.1 (5)	C36—C37—C38—C33	1.0 (4)
C12—C11—C16—C15	−0.2 (4)	O2—C10—N1—C5	9.2 (5)
C9—C11—C16—C15	−179.9 (2)	C9—C10—N1—C5	−170.7 (2)
C14—C15—C16—C11	0.7 (5)	O2—C10—N1—C8	−172.1 (3)
N1—C8—C17—O3	−164.49 (16)	C9—C10—N1—C8	7.93 (19)
C9—C8—C17—O3	−67.2 (2)	C4—C5—N1—C10	139.2 (3)
N1—C8—C17—C18	75.8 (2)	C6—C5—N1—C10	−40.5 (4)
C9—C8—C17—C18	173.01 (18)	C4—C5—N1—C8	−39.0 (4)
O3—C17—C18—C32	135.15 (18)	C6—C5—N1—C8	141.3 (2)
C8—C17—C18—C32	−102.8 (2)	C17—C8—N1—C10	109.6 (2)
O3—C17—C18—C19	10.6 (2)	C9—C8—N1—C10	−7.72 (19)
C8—C17—C18—C19	132.61 (18)	C17—C8—N1—C5	−71.7 (3)
C32—C18—C19—N2	−155.04 (19)	C9—C8—N1—C5	170.9 (2)
C17—C18—C19—N2	−31.6 (2)	C25—C20—N2—O3	31.7 (3)
C32—C18—C19—C26	−32.9 (3)	C21—C20—N2—O3	−154.0 (2)
C17—C18—C19—C26	90.6 (2)	C25—C20—N2—C19	149.9 (2)
C25—C20—C21—C22	−1.6 (4)	C21—C20—N2—C19	−35.8 (3)
N2—C20—C21—C22	−176.0 (2)	C26—C19—N2—C20	156.22 (18)
C20—C21—C22—C23	0.4 (4)	C18—C19—N2—C20	−80.1 (2)
C21—C22—C23—C24	0.7 (5)	C26—C19—N2—O3	−82.5 (2)
C22—C23—C24—C25	−0.5 (5)	C18—C19—N2—O3	41.1 (2)
C23—C24—C25—C20	−0.8 (4)	C3—C2—O1—C1	11.6 (5)
C21—C20—C25—C24	1.9 (4)	C7—C2—O1—C1	−167.9 (3)
N2—C20—C25—C24	176.3 (2)	C20—N2—O3—C17	90.60 (19)
N2—C19—C26—C27	30.9 (3)	C19—N2—O3—C17	−35.8 (2)
C18—C19—C26—C27	−85.9 (3)	C8—C17—O3—N2	−109.89 (18)
N2—C19—C26—C31	−149.3 (2)	C18—C17—O3—N2	14.6 (2)

### Hydrogen-bond geometry (Å, º)

**Table:**

*D*—H···*A*	*D*—H	H···*A*	*D*···*A*	*D*—H···*A*
C19—H19···O2^i^	0.98	2.41	3.338 (3)	158
C21—H21···O2^i^	0.93	2.50	3.425 (4)	173
C37—H37···O1^ii^	0.93	2.58	3.407 (4)	148

Symmetry codes: (i) *x*−1/2, −*y*+1/2, *z*−1/2; (ii) −*x*, −*y*+1, −*z*+2.
